# Contribution of Fluorescence Techniques in Determining the Efficiency of the Non-thermal Plasma Treatment

**DOI:** 10.3389/fmicb.2018.02171

**Published:** 2018-09-10

**Authors:** Gaëlle Carré, Emilie Charpentier, Sandra Audonnet, Christine Terryn, Mohamed Boudifa, Christelle Doliwa, Zouhaier Ben Belgacem, Sophie C. Gangloff, Marie-Paule Gelle

**Affiliations:** ^1^Laboratoire de Biomatériaux et Inflammation en Site Osseux (EA 4691), SFR CAP-Santé, FED 4231, Université de Reims Champagne-Ardenne, Reims, France; ^2^Unité de Formation et de Recherche de Pharmacie, Université de Reims Champagne-Ardenne, Reims, France; ^3^URCACyt – Plateau technique de cytométrie en flux, Université de Reims Champagne-Ardenne, Reims, France; ^4^PICT – Plateforme d’Imagerie Cellulaire et Tissulaire, Université de Reims Champagne-Ardenne, Reims, France; ^5^CRITT-MDTS, Charleville-Mézières, France; ^6^UFR Odontologie, Reims, France; ^7^Pôle Médecine Bucco-Dentaire, Centre Hospitalier Universitaire de Reims, Reims, France

**Keywords:** non-thermal plasma, *Staphylococcus aureus*, fluorescence, flow cytometry, confocal microscopy

## Abstract

We have recently developed a non-thermal plasma (NTP) equipment intended to sterilize fragile medical devices and maintain the sterile state of items downstream the treatment. With traditional counts on agar plate a six log reduction of *Staphylococcus aureus* viability was obtained within 120 min of O_2_, Ar, or N_2_ NTP treatments. However to determine the best NTP process, we studied the different physiological states of *S. aureus* by flow cytometry (FC) and confocal laser scanning microscopy (CLSM) focusing on the esterasic activity and membrane integrity of the bacteria. Two fluorochromes, 5-(and-6)-carboxy-2^′^,7^′^-dichlorofluorescein diacetate and propidium iodide were used in order to distinguish three sub-populations: metabolically active, permeabilized, and damaged bacteria that can be in the viable but nonculturable state. FC and CLSM highlight that O_2_ and Ar NTP treatments were the most attractive processes. Indeed, a 5 min of Ar NTP generated a high destruction of the structure of bacteria and a 120 min of O_2_ NTP treatment led to the higher decrease of the total damaged bacteria population. SEM observations showed that in presence of clusters, bacteria of upper layers are easily altered compared to bacteria in the deeper layers. In conclusion, the plate counting method is not sufficient by itself to determine the best NTP treatment. FC and CLSM represent attractive indicator techniques to select the most efficient gas NTP treatment generating the lowest proportion of viable bacteria and the most debris.

## Introduction

A recent interest for non-thermal plasma (NTP) processes has raised in the medical field in order to sterilize fragile devices that do not support conventional techniques such as high pressure saturated steam, ethylene oxide treatment, and ionizing radiation (Moisan et al., 2001; Burts et al., 2009; Hauser et al., 2011; Scholtz et al., 2015).

Non-thermal plasma is an ionized gas consisting of UV-VUV photons, charged particles, reactive species (e.g., reactive oxygen species – ROS, OH., O_2_, ^1^O_2_^-^; reactive nitrogen species – RNS, NO^∙^, ONOO^∙^, …), excited molecules (e.g., excited O_2_, N_2_, …) depending on the plasma process and the gas nature (Moisan et al., 2001; Laroussi and Leipold, 2004; Gaunt et al., 2006; Liao et al., 2017). Nowadays there are numerous NTP techniques such as corona discharge, electric barrier discharge, microwave discharge or plasma jet that can inactivate various bacterial strains (Lerouge et al., 2001; Moisan et al., 2001; Boscariol et al., 2008; Sureshkumar et al., 2010; Fröhling et al., 2012; Kvam et al., 2012; Alkawareek et al., 2014; Fröhling and Schlüter, 2015; Scholtz et al., 2015; Impe et al., 2018). These technologies differ from each other in terms of electrical current, discharge reactor designs, working pressures, and operating conditions (nature and flow rate of the gas).

To meet the sterilization standards such as maintaining the sterile state of medical devices after the end of the process, we have developed a new technique able to get NTP inside a sealed bag while maintaining the integrity of bag membranes (Popot and Gelle, 2012). We have recently demonstrated with the plate counting method that our NTP treatment process was highly effective against *Bacillus subtilis* spores or vegetative bacteria such as *Pseudomonas aeruginosa* and *Staphylococcus aureus* (Ben Belgacem et al., 2017). However if the plate counting method is a gold standard to evaluate a sterilization process, this approach does not take into account the different physiological states of bacteria.

Indeed since the 1970s, numerous authors have demonstrated that bacteria can adapt to their environment presenting different states of viability including viable but nonculturable (VBNC) state (Oliver, 2010; Ramamurthy et al., 2014). A cell is usually considered alive when it is metabolically active and capable of reproduction with intact cell membranes (Emerson et al., 2017). VBNC bacteria are alive but they do not divide using conventional culturing technique. However, they can grow and divide again when culture conditions are more favorable for their metabolic recovery as a consequence the bacteria can thereafter express their pathogenicity again (Emerson et al., 2017). Thus VBNC bacteria represent a significant risk in many domains such as medical field (Passerat et al., 2009; Dinu and Bach, 2011; Zandri et al., 2012; Mali et al., 2017; Lee and Bae, 2017).

Recently, different techniques have been developed to study the bacterial different viability states in order to evaluate the efficiency of antimicrobial treatments. These techniques are based on PCR and (RT)-PCR assays (Stanley et al., 2007; Emerson et al., 2017), Fourier transform infrared spectroscopy (FTIR) (Meng et al., 2016) or fluorescence methods (Khan et al., 2010; Sträuber and Müller, 2010; Pasquaroli et al., 2013; Ambriz-Aviña et al., 2014; Li et al., 2017) such as epifluorescence microscopy, flow cytometry (FC) and confocal laser scanning microscopy (CLSM). FC technique represents a fast way to determine the physiological state of bacteria in suspension (Ou et al., 2017). CLSM is a powerful approach for high-resolution imaging of fluorescent bacteria on different focal planes. These fluorescence techniques highlighted some altered characteristics of bacteria such as membrane integrity, pump activity, membrane potential, or metabolic activity: respiratory and enzymatic activities. These methods are highly compatible with a broad range of fluorescent dyes and cell labeling (Léonard et al., 2016).

*Staphylococcus aureus* is a well-known human pathogen that causes a wide range of clinical infections such as endocarditis, osteoarticular, skin and soft tissue, or medical device-related infections and shock syndrome leading to the death of patients (Burts et al., 2009; Josse et al., 2015; Tong et al., 2015; Kahl et al., 2016; Reddy et al., 2017).

In a previous study, we have demonstrated with culture plating method that *S. aureus* can be inactivated in 120 min by O_2_, Ar, or N_2_ NTP treatments according to the European sterilization norm (Ben Belgacem et al., 2017). The aim of this study, was to control the efficiency of our O_2_, Ar, and N_2_ NTP treatments using plate counting, FC, CLSM, and scanning electron microscopy (SEM) observations to investigate more precisely *S. aureus* viability states.

## Materials and Methods

### *Staphylococcus aureus* Strains, Growth Conditions

*Staphylococcus aureus* CIP 53.154 were provided from Pasteur Institute (Paris, France). Bacteria were stored at -80°C in an appropriate medium supplemented with 50% glycerol. The strain was sequentially subcultured three times in a specific nutrient medium containing glucose, peptone from casein and soybean as well as sodium chloride. The first preculture was carried out at 37°C for 18 h after thawing 1 mL of the cryotube added to 10 mL of nutrient medium. Four milliliters from the previous culture were transferred to a fresh culture medium (50 mL) and incubated for 7 h at 37°C in a rotary shaker at a constant speed of 250 × *g*. The final bacterial suspension was made by transferring 1 mL from the second culture to a fresh culture medium (50 mL) and incubating for 18 h at 37°C.

Bacteria were centrifuged at 4,200 ×*g* for 10 min at 4°C and washed twice with distilled water. After the ultimate wash, the bacteria were re-suspended in distilled water to achieve a concentration of approximately 10^8^ colony forming units (CFU)/mL. Twenty microliters of this bacterial suspension were deposited on glass slides and then dried for 10 min before plasma treatment as previously described (Ben Belgacem et al., 2017).

### Sterilization by Non-thermal Plasma

The prototype manufactured by Sominex (Bayeux, France) consisted of a stainless steel vacuum chamber 35 L (Popot and Gelle, 2012). A high vacuum state was obtained by using two pumps (Agilent TRISCROLL 300 and Agilent V301). The plasma discharge was generated directly inside a sealed bag (SüdPack^®^Medica, Germany) by a radio-frequency (RF) polarization plate (Ø 300 mm; RF generator: 13.56 MHz; 300 W) coupled with two magnetic coils (0–14 Gauss; SEF, Labege, France) located at the top and the bottom of the vacuum chamber (Popot and Gelle, 2012; Ben Belgacem et al., 2017). Three mass flow controllers were connected to the gas lines (O_2_, Ar, and N_2_) to control the flow rate (0.5 sccm). Contaminated glass slides were placed inside the bag, which was sealed and then set on the RF polarization plate. When the low pressure reached 1.45 × 10^-4^ mbar, the gases were injected (0.5 sccm) through the first Tyvek^®^membrane and the excess gas was released through the second Tyvek^®^membrane into the vacuum chamber. Then, the discharge was induced by the RF polarization (25 W) inside the bag and the plasma was densified by the magnetic field (14 Gauss) for 5, 15, and 120 min. Controlling the pressure difference between the vacuum chamber and the bag, the plasma is kept confined inside the bag.

At the end of the process, the pumping system was stopped and nitrogen was injected into the vacuum chamber until the system returned to atmospheric pressure. To evaluate the antimicrobial efficiency, untreated bacteria were used as controls for each independent experimentations. These controls consisted of non-exposed samples to plasma as well as samples exposed only to low pressure.

### Counts on Agar Plate

For each gas plasma treatment and each time period, seven independent experimentations were performed. Glass slides were submitted to mechanical agitation [1 min of vortex, 1 min in a ultrasonic bath (VWR TH USC 300 THD, 45 KHz), and 1 min of vortex] in 15 mL distilled water to detach bacteria and obtain homogenous suspensions. Both bacterial dilution and seeding on nutrient agar plates (trypto-casein soy agar, Biokar, France) were performed with EasySpiral Pro^®^ (Interscience, France). After 24 h of incubation at 37°C, the colonies on agar plates were counted with the Scan^®^1200 (Interscience, France). A second count was made after 48 h to verify if new colonies could be visualized.

### Fluorescent Dye Preparation

Live and dead heat-treated (15 min at 80°C) bacteria used as controls as well as bacterial samples were stained with 5-(and-6)-carboxy-2^′^,7^′^-dichlorofluorescein diacetate (DCFDA) and propidium iodide (PI). The stock solution at 9.4 mM of carboxy-DCFDA (C369, ThermoFisher Scientific) was prepared by dissolving the dye in acetone and stored at -20°C in the dark. The PI (Sigma-Aldrich Co., United States) was dissolved in milliQ water in order to have a 0.7 mM stock solution that was kept at -20°C in the dark.

### Flow Cytometric Analysis

#### Staining Procedure

Live and dead heat-treated bacteria (15 min at 80°C) were used as controls to optimize the staining procedure. As described above, glass slides were immersed in 15 mL of distilled water, sonicated and vortexed to obtain bacterial suspensions. One milliliter of each suspension was introduced in a Trucount tube (Becton Dickinson, United States) containing 50,950 beads to accurately collect the same volume at each FC acquisition and thereby allow the calculation of the bacterial concentration per mL (GuoHui et al., 2013).

The bacterial suspension was incubated with 19 μM of DCFDA for 15 min at 37°C to allow deacetylation of DCFDA by esterases into a fluorescent derivative, the dichlorofluorescein (DCF). Ten min after DCFDA, PI (0.75 μM) was added to the mix to allow labeling of permeabilized membranes of bacteria. Stained samples were kept in the dark for no more than 30 min until flow cytometric analysis was performed.

#### Flow Cytometric Measurement

Analyses were performed with a LSR Fortessa^TM^ (Becton Dickinson, United States) flow cytometer. Excitations of DCFDA and PI were done at wavelengths of 488 and 561 nm, respectively, while fluorescence emissions were detected using band pass filters of 530/30 and 610/20 nm, respectively. No compensation settings were applied. Trucount beads were excited at wavelength of 640 nm and emission was detected using band pass filter of 670/14 nm. All recorded signals were logarithmically amplified. Gates created in the dot-plot FSC(H) vs. SSC(H) were preset to identify bacterial and beads populations. The bacterial population was gated using the side scatter (SSC) and forward scatter (FSC) parameters to dissociate it from the background signal and debris. The fluorescent dot plots were gated from this bacterial population. Data acquisition was set to 1,000 beads at a low flow rate (12 μL/min) corresponding to 19.6 μL of the sample. Seven independent assays were performed for each condition. The software FlowJo^®^ (FlowJo, Oregon, United States) was used to analyze FC data.

### Confocal Laser Scanning Microscopy

#### Staining Procedure

Mix DCFDA (470 μM final concentration) and PI (35 μM final concentration) were prepared extemporaneously in 40 μL of distilled water. This stained solution was deposited on controls or NTP treatments glasses slides, covered with a cover slip, and sealed with a clear varnish to prevent drying of the bacterial sample during visualization with the CLSM.

#### Images Acquisition

Immunofluorescence-labeled bacteria preparations were studied using a Zeiss LSM 710^®^ NLO confocal laser scanning microscope (Carl ZEISS SAS, Marly le Roi, France) with the 20× objective (ON 0.8) and numerical zoom of 2. DCFDA and PI were simultaneously excited by 488 nm line of argon laser and diode laser 561 nm. Emitted signals were, respectively, collected with 505–544 nm and 600–700 nm bandpass filters. Two images from 10 mm^2^ sample areas were acquired for each condition. A minimum of triplicate experiments were done for each condition.

#### Images Analysis and Quantification

Quantification of images was performed using a home-made macro on ImageJ software (National Institute of Health, United States). The macro steps included intensity threshold and count of metabolically active and permeabilized bacteria using “particle analysis” with shape parameter on 505–544 nm and 600–700 nm images, respectively. The number of bacteria was then divided by the image area size to obtain a density of events. Then, the damaged number of events was calculated by counting bacteria on logic AND image between the two acquired images.

### Scanning Electron Microscopy

Contaminated glass slides before or after the different NTP treatments were fixed with 2.5% glutaraldehyde in phosphate buffer solution (PBS) for 1 h at room temperature, and rinsed twice with PBS for 10 min. The samples were than dehydrated in graded series of ethanol/water solutions at 50, 70, 90, and 100% (twice) and covered with hexamethyldisilazane solution (Sigma-Aldrich, St. Louis, MO, United States) (Braux et al., 2011). The samples were dessicated overnight at room temperature then coated with platinum on a Jeol Ultrafine Coat JUC 5000 and observed on a FEG Scanning Electron Microscope Zeiss Ultra Plus (Ibisa Platform of Electron Microscopy, Tours, France). Two independent assays were performed for each condition.

### Statistical Analysis

Statistical analysis was performed by the Wilcoxon’s *t* test (Prism 5, GraphPad Software, San Diego, CA, United States). A value of ^∗^*p* < 0.05, ^∗∗^*p* < 0.01, ^∗∗∗^*p* < 0.001 was considered to be statistically significant.

## Results

### Efficiency of Non-thermal Gas Plasma Against *S. aureus* Determined by the Plate Counting Method

**FIGURE 1:**
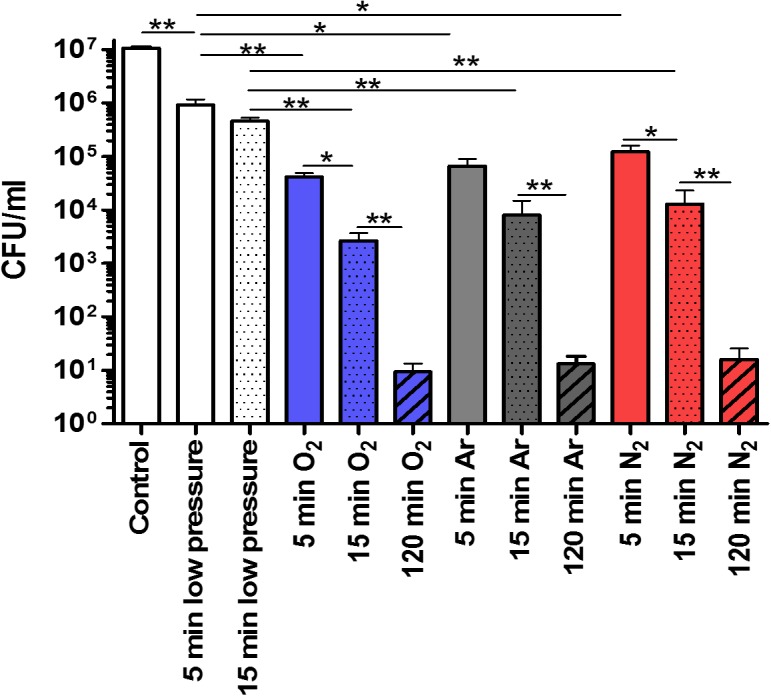
Inactivation of *Staphylococcus aureus* with N_2_, O_2_, Ar NTP (25 W; 14 G; and 0.5 sccm) determined by counting on agar plate. Error bars represent the standard error of the mean from seven experiments in triplicate. Statistical analysis was performed by the Wilcoxon test ^∗^*p* < 0.05; ^∗∗^*p* < 0.01.

Plate counts, a gold standard method highlighting only the bacteria capable to grow on agar plate, was used to quantify the biocidal efficiency of different NTP treatments. The bactericidal effect on *S. aureus* was measured by comparing the number of CFUs in the plasma treated sample with the control (1.07 × 10^7^ CFU/mL) (**Figure 1**).

When samples were subjected only to the low pressure, a significant decrease in *S. aureus* viability was observed after 5 min (9.24 × 10^5^ CFU/mL, *p* < 0.01). However, after 15 min of low pressure no statistical significant decrease was observed as compared to 5 min with a recovery of 4.62 × 10^5^ CFU/mL.

Looking at the effects of 5 min of O_2_, Ar, or N_2_ NTP treatments, we observed each time a significantly higher reduction of *S. aureus* viability than in presence of only low pressure treatment (**Figure 1**, O_2_, *p* < 0.01; Ar, *p* < 0.05; N_2_, *p* < 0.05). Furthermore, the effectiveness of all NTP treatments increased as a function of the time. After 15 min of NTP treatment, O_2_ treatment tended to be more effective (2.67 × 10^3^ CFU/mL) as compared to Ar (8 × 10^3^ CFU/mL) and N_2_ (1.29 × 10^4^ CFU/mL) NTP treatments. However, after 120 min of NTP treatments, the bactericidal effect (6 log reduction) was obtained with all the three gases (O_2_: 9.38 × 10^0^ CFU/mL; Ar: 1.32 × 10^1^ CFU/mL; N_2_: 1.6 × 10^1^ CFU/mL).

### Efficacy of Non-thermal Gas Plasma Against *S. aureus* Determined by FC

**FIGURE 2:**
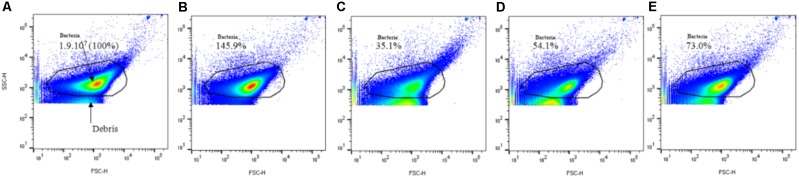
Selection of the *Staphylococcus aureus* population by FC based on FSC and SSC parameters. Effect of 15 min of NTP treatments. **(A)** Control: number of events/mL; **(B)** 15 min low pressure; **(C)** 15 min O_2_; **(D)** 15 min Ar; **(E)** 15 min N_2_. **(B–E)** Expressed as a percentage of the control population.

To assess the different physiological states of the *S. aureus* population after short NTP exposures, the samples were analyzed by FC. **Figure 2** shows an example of the bacterial population detected in the gate. This is a heterogeneous control population (1.9 × 10^7^ events/mL = 100%) composed of bacteria with different sizes and granularities.

After 15 min of low pressure, this population seemed to increase (145.9% of the bacterial population control) (**Figures 2A,B**). After 15 min of NTP treatments, with each gas, the bacterial population density decreased (O_2_: 35.1%; Ar: 54.1%; N_2_: 73%) (**Figures 2C–E**).

**FIGURE 3:**
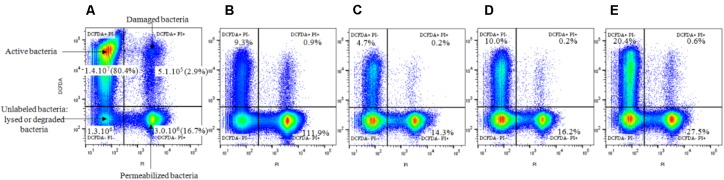
Influence of the gas nature (N_2_, O_2_, and Ar) after 15 min of NTP treatments on *S. aureus* physiological states, determined by FC. The fluorescent dot plots were gated from the bacterial population previously determined and were obtained in response to double-staining with DCFDA and PI. **(A)** Control: total labeled events (1.93 × 10^7^ events/mL = 100%); **(B)** 15 min low pressure; **(C)** 15 min O_2_; **(D)** 15 min Ar; **(E)** 15 min N_2_. **(B–E)** Expressed as a percentage of the total labeled events of the control.

From the previous gate, bacteria population presenting esterasic activities and/or permeabilized membrane was analyzed after 15 min of NTP treatments. As seen in **Figure 3**, bacteria populations were divided in four distinct sub-populations based on their differential PI and DCFDA staining characteristics. These sub-populations consisted of: metabolically active bacteria (upper left quadrant; DCFDA+ and PI-); damaged bacteria with esterasic activity and permeabilized membrane (upper right quadrant; both DCFDA+ and PI+); permeabilized bacteria without esterasic activity (lower right quadrant; DCFDA- and PI+); and unlabeled population corresponding to lysed or degraded bacteria (lower left quadrant; both DCFDA- and PI-). The whole three labeled sub-populations (active, damaged, and permeabilized bacteria; 1.78.10^7^ events/mL) represented the labeled bacteria control.

Active bacteria (1.4 × 10^7^ events/mL) represented the major labeled sub-population (80.4% of the labeled bacteria control), whereas the other sub-populations were smaller: 5.1 × 10^5^ events/mL (2.9%) for damaged bacteria, 3.0 × 10^6^ events/mL (16.7%) for permeabilized bacteria and 1.3 × 10^6^ events/mL for unlabeled population (**Figure 3A**).

Low pressure treatment of 15 min increased the permeabilized bacteria population to 111.9% of the labeled bacteria control, essentially by decreasing the active bacteria population (from 80.4 to 9.3%) (**Figure 3B**). After 15 min of NTP treatments, a decrease of the active bacteria population (O_2_: 4.7%; Ar: 10%; N_2_: 20.4%) was observed for the benefit of the permeabilized bacterial population (O_2_: 14.3%; Ar: 16.2%; N_2_: 27.5%) and unlabeled bacteria or debris (**Figures 3C–E**).

Based on the total gated population, **Table 1** and **Supplementary Figure S1** show the distribution of the three sub-populations (active, damaged, and permeabilized) after 5 and 15 min of treatments (*n* = 7). The labeled population in the control (1.93 × 10^7^ events/mL) was composed of 72.36% active, 3.21% damaged, and 24.44% permeabilized bacteria. Looking more in details, the different treatments (low pressure and NTP), systematically induced a reduction of the whole labeled populations and modified their repartitions.

**Table 1:** Analyse by FC of *Staphylococcus aureus* sub-populations [total labeled, metabolically active sub-population (DCFDA+ and PI-), damaged sub-population (DCFDA+ and PI+), and permeabilized sub-population (DCFDA- and PI+)] labeled with PI and DCFDA fluorochromes after 5 and 15 min of O_2_, Ar, and N_2_ NTP treatments.

	Number of events/mL				% of the total labeled control			
	Total labeled events	Active (DCFDA+ and PI-)	Damaged (DCFDA+ and PI+)	Permeabilized (DCFDA- and PI+)	Total labeled events	Active (DCFDA+ and PI-)	Damaged (DCFDA+ and PI+)	Permeabilized (DCFDA- and PI+)
Control	1.93E + 07 ± 5.25E + 06	1.40E + 07 ± 5.60E + 06	6.19E + 05 ± 2.68E + 05	4.72E + 06 ± 1.92E + 06	100.00	72.36	3.21	24.44
5 min low pressure	1.27E + 07 ± 5.93E + 06	1.34E + 0 ± 9.11E + 05	2.09E + 05 ± 1.34E + 05	1.12E + 07 ± 6.10E + 06	65.87	6.95	1.08	57.84
15 min low pressure	1.65E + 07 ± 6.99E + 06	1.72E + 06 ± 1.60E + 06	1.97E + 05 ± 1.63E + 05	1.45E + 07 ± 6.72E + 06	85.27	8.92	1.02	75.33
5 min O_2_	6.65E + 06 ± 5.16E + 06	8.01E + 05 ± 6.20E + 05	6.11E + 04 ± 4.67E + 04	5.79E + 06 ± 4.64E + 06	34.43	4.14	0.32	29.97
15 min O_2_	2.26E + 06 ± 1.69E + 06	5.01E + 05 ± 4.88E + 05	3.73E + 04 ± 2.64E + 04	1.72E + 06 ± 1.35E + 06	11.68	2.59	0.19	8.89
5 min Ar	4.61E + 06 ± 2.85E + 06	1.25E + 06 ± 9.05E + 05	1.11E + 05 ± 1.08E + 05	3.25E + 06 ± 1.92E + 06	23.86	6.48	0.58	16.80
15 min Ar	2.01E + 06 ± 1.18E + 06	5.76E + 05 ± 4.88E + 05	4.58E + 04 ± 2.30E + 04	1.39E + 06 ± 7.59E + 05	10.43	2.98	0.24	7.21
5 min N_2_	1.93E + 07 ± 5.25E + 06	1.40E + 07 ± 5.60E + 06	1.93E + 07 ± 5.25E + 06	1.93E + 07 ± 5.25E + 06	54.57	4.91	0.44	49.22
15 min N_2_	2.43E + 07 ± 2.50E + 06	6.45E + 05 ± 1.14E + 06	4.36E + 04 ± 3.28E + 04	1.74E + 06 ± 1.41E + 06	12.58	3.34	0.23	9.01

*Events per mL are means of seven measurements ± standard deviation. These data are then expressed as percentages of the total labeled events of the control*.

Low pressure treatments decreased active bacteria population to 8.92% after a 15 min of treatment and in the meantime increased the permeabilized bacteria population to 75.33%. NTP treatments induced a time-dependent decrease of the active and permeabilized bacteria populations, whatever the gas used. After 5 min of treatments, Ar plasma led to the highest decrease of the total labeled population (23.86%) compared to O_2_ (34.43%) and N_2_ (54.57%). After 15 min of NTP treatments, this decline was enhanced (Ar: 10.43%; O_2_: 11.68%; N_2_: 12.58%). The 15 min of O_2_ NTP treatment was the most efficient in regard to the bacteria populations with esterasic activity (2.78%), compared to the others NTP treatments (Ar: 3.22%; N_2_: 3.57%).

Beyond 15 min of NTP treatments, the remaining labeled bacteria populations were too low and may have been drowned in the background noise. Therefore, the FC analysis was not suitable.

### Efficiency of NTP Plasma Against *S. aureus* Determined by CLSM

**FIGURE 4:**
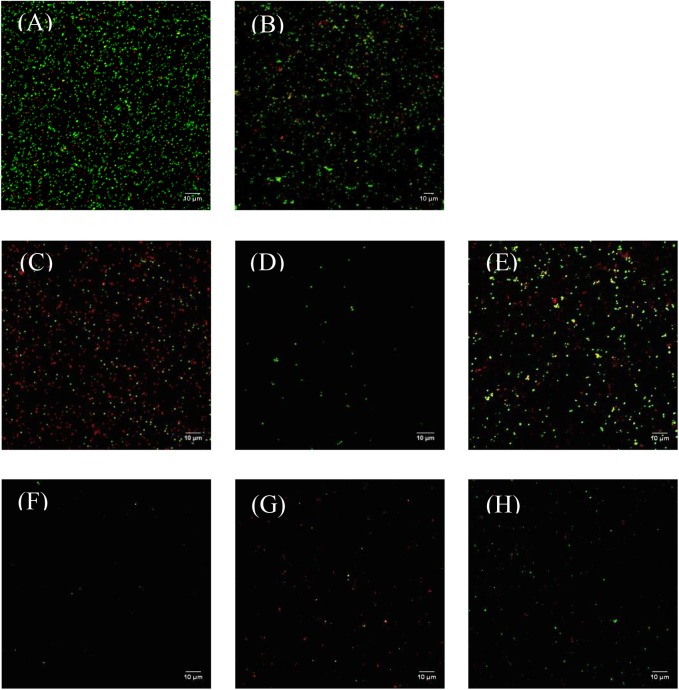
Representative CLSM images of *Staphylococcus aureus* labeled with PI and DCFDA fluorochromes after 5 or 120 min of O_2_, Ar, or N_2_ NTP treatments. DCFDA+ and PI– bacteria (*green*). DCFDA+ and PI+ bacteria (*yellow*). DCFDA– and PI+ bacteria (*red*). **(A)** Control; **(B)** 5 min low pressure; **(C)** 5 min O_2_; **(D)** 5 min Ar; **(E)** 5 min N_2_; **(F)** 120 min O_2_; **(G)** 120 min Ar; **(H)** 120 min N_2_.

Confocal laser scanning microscopy approach is complementary to the FC as it allows to evaluate low density of bacteria such as active (DCFDA+ and PI-; green event), damaged (DCFDA+ and PI+; yellow event), and permeabilized (DCFDA- and PI+; red event) sub-populations (**Figure 4**, **Table 2**, and **Supplementary Figure S2**). The control population was composed of 64.85% active bacteria and only 12.38% permeabilized or 22.72% damaged bacteria (**Figure 4A** and **Table 2**).

**Table 2:** Density of *Staphylococcus aureus* population determined by CLSM with DCFDA and PI after low pressure for 5 min, O_2_, Ar, and N_2_ NTP treatments for 5 and 120 min (events/mm^2^).

	Number of events (detected for 1 mm^2^)				% of the total labeled control			
	Total labeled events	Active (DCFDA+ and PI-)	Damaged (DCFDA+ and PI+)	Permeabilized (DCFDA- and PI+)	Total labeled events	Active (DCFDA+ and PI-)	Damaged (DCFDA+ and PI+)	Permeabilized (DCFDA- and PI+)
Control	7.69E + 04	4.99E + 04	1.75E + 04	9.52E + 03	100.00	64.85	22.72	12.37
5 min low pressure	1.09E + 04	8.07E + 03	1.02E + 03	1.94E + 03	14.19	10.50	1.33	2.52
5 min O_2_	1.61E + 04	7.45E + 03	2.27E + 03	6.20E + 03	20.98	9.69	2.96	8.07
5 min Ar	3.37E + 03	2.40E + 03	7.05E + 02	2.69E + 02	4.39	3.12	0.92	0.35
5 min N_2_	2.91E + 04	1.46E + 04	4.64E + 03	9.78E + 03	37.80	19.02	6.03	12.72
120 min O_2_	8.83E + 02	4.83E + 02	1.70E + 01	3.82E + 02	1.15	0.63	0.02	0.50
120 min Ar	3.04E + 03	4.20E + 02	1.02E + 03	2.25E + 03	3.95	0.55	1.33	2.93
120 min N_2_	3.57E + 03	1.68E + 03	4.34E + 02	1.46E + 03	4.65	2.18	0.56	1.90

*Evaluation of the total density in events/mm^*2*^ of total labeled bacteria, metabolically active bacteria (DCFDA+ and PI-), damaged bacteria (DCFDA+ and PI+) and permeabilized bacteria (DCFDA- and PI+) and percentage of these labeled populations in comparison to control (*n* ≥ 3)*.

After 5 min of treatments (low pressure and NTP), a decrease of the whole labeled populations was observed and it appeared clearly that the Ar NTP treatment generated the highest decrease in bacterial density (>95%, **Figure 4D** and **Table 2**). Furthermore, this total decrease was not linked to an increase of permeabilized or damaged bacteria populations, suggesting that the bacteria were completely destroyed, making impossible the labeling with PI.

To obtain a bacterial viability reduction of 6 log, the plasma process had to be extended to 120 min according to the plate counting method (**Figure 1**). In these conditions, applying solely low pressure that decreased up to 10^-5^ mbar was not relevant as it was only reaching 1.45 × 10^-4^ mbar during the NTP treatments. Whatever 120 min of NTP treatments, a reduction of almost 95% of the total labeled population could be observed (**Figures 4F–H** and **Table 2**). However, the O_2_ NTP treatment was the most efficient (0.63% active bacteria; 0.50% permeabilized bacteria; and 0.02% damaged bacteria). Like in the FC analysis, the N_2_ NTP treatment was the least efficient (2.18% active bacteria, 0.56% damaged bacteria, and 1.90% permeabilized bacteria).

### Plasma Effect on *S. aureus* Morphology Determined by SEM

**FIGURE 5:**
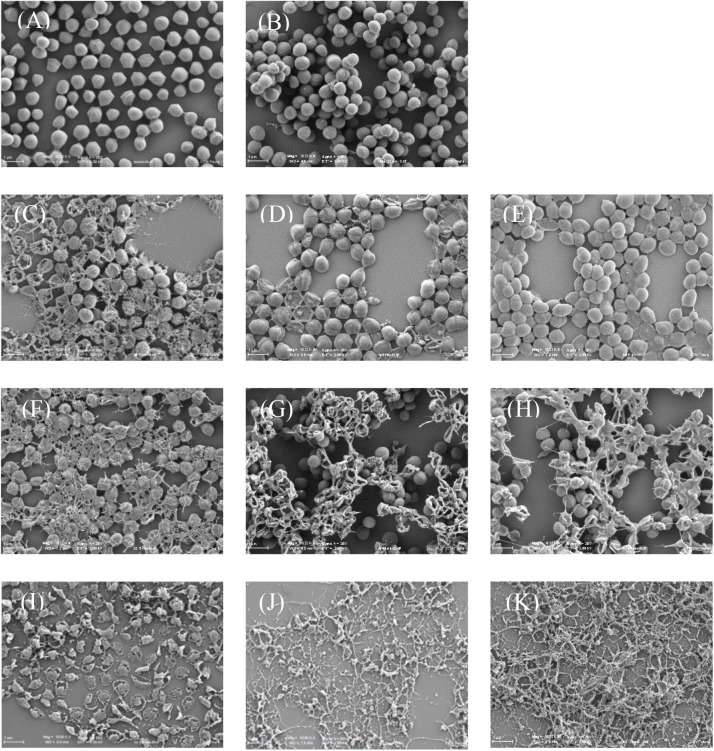
Representative SEM images of *Staphylococcus aureus* before and after plasma treatments (G × 10,000). **(A)** Control; **(B)** 120 min low pressure; **(C)** 5 min O_2_; **(D)** 5 min Ar; **(E)** 5 min N_2_; **(F)** 120 min O_2_; **(G)** 15 min Ar; **(H)** 15 min N_2_; **(I)** 15 min O_2_; **(J)** 120 min Ar; and **(K)** 120 min N_2_.

The observation by SEM confirmed that *S. aureus* had a round shape (**Figure 5A**) and that 120 min of low pressure exposure at 10^-5^ mbar did not lead to major bacteria alterations (**Figure 5B**). After 5 min exposure if a large proportion of bacteria were undamaged with the N_2_ NTP treatment (**Figure 5E**), O_2_ and Ar NTP treatments led to some cell alterations such as rupture of membranes and loss of intracellular material (**Figures 5C,D**). After 15 min exposure, NTP treatments were more effective (**Figures 5F–H**). However if they were more debris, the bacteria located in the deepest layers of the deposit seemed to be protected, keeping their round shape (**Figures 5G,H**). After 120 min whatever NTP treatments, there were mainly bacterial debris and only very few bacteria could still be visualized (**Figures 5I–K**).

## Discussion

The aim of this study was to control the efficiency of our O_2_, N_2_, and Ar NTP process by two fluorescent techniques (FC and CLSM). These techniques allow to highlight more precisely *S. aureus* viability states compared to standard culture plating method.

Recently, we have demonstrated through the plate counting method that O_2_, Ar, or N_2_ NTP process could inactivate *S. aureus* within 120 min, which is line with the European norm of sterilization (Ben Belgacem et al., 2017). This technique highlights only bacteria dividing and not the damaged bacteria, such as VBNC, that are capable to divide and express their pathogenicity when conditions are more favorable thereafter. In literature, *S. aureus* can enter into VBNC or dormancy states when subjected to environmental stresses such as short NTP exposure (Abramzon et al., 2006; Vandervoort et al., 2008; Joaquin et al., 2009). VBNC are considered as a threat to public health due to their non-detectability by conventional methods in the medical field (Oliver, 2010; Zandri et al., 2012; Pasquaroli et al., 2013; Gonçalves and de Carvalho, 2016).

Flow cytometry (a quantitative method) and CLSM (a semi-quantitative method) analyses allowed to characterize the physiological states of *S. aureus* (active, permeabilized, and damaged bacteria) after NTP treatments. Two probes were used: PI staining bacterial DNA when the cell membranes are permeabilized and DCFDA revealing the esterasic activity of bacteria.

Our process requires working at low pressure before inducing a plasma in a sealed bag. Therefore we have determined the effect of low pressure on *S. aureus*. Although it did not allow to meet the sterility standards by itself according to culture plating method (Ben Belgacem et al., 2017), some bacterial alterations such as a decrease of esterasic activity and an increase of membranes permeabilization have been observed. Indeed, it can be suggested that bacteria have been weakened by low pressure unlike *B. subtilis* spores in the same process conditions (Ben Belgacem et al., 2017).

Concerning the O_2_, N_2_, and Ar NTP treatments, a time-dependent decrease of cultivable bacteria population was observed, up to 6 log reduction after 120 min of treatment. Similarly both fluorescent methods revealed a time-dependent decrease of the total labeled bacteria. Nonetheless, these techniques highlighted some differences between the three bacteria sub-populations repartition depending on the NTP treatments. They suggested that a short time of Ar NTP treatment was more efficient in the alteration of bacteria and the production of the lysed bacteria or debris. After 15 min and whatever the gas used, the efficiency of the treatments was enhanced, as underlined with FC or plate counting method. However, no significant difference in the sub-populations repartition was observed by FC whatever the NTP treatments.

Although the FC provides a quick analysis of different physiological states there are some limitations for its use, e.g., the low remaining numbers of events that can be labeled may be drowned in the background noise. Consequently, CLSM has been used to highlight the different physiological states at the end of process. CLSM revealed that a 120 min of O_2_ or Ar NTP treatments led to the smaller proportion of active bacteria compared to N_2_ treatment. Nevertheless O_2_ plasma treatment can be considered as the most interesting while reducing significantly the number of damaged *S. aureus* that could have been potential VBNC bacteria.

**FIGURE 6:**
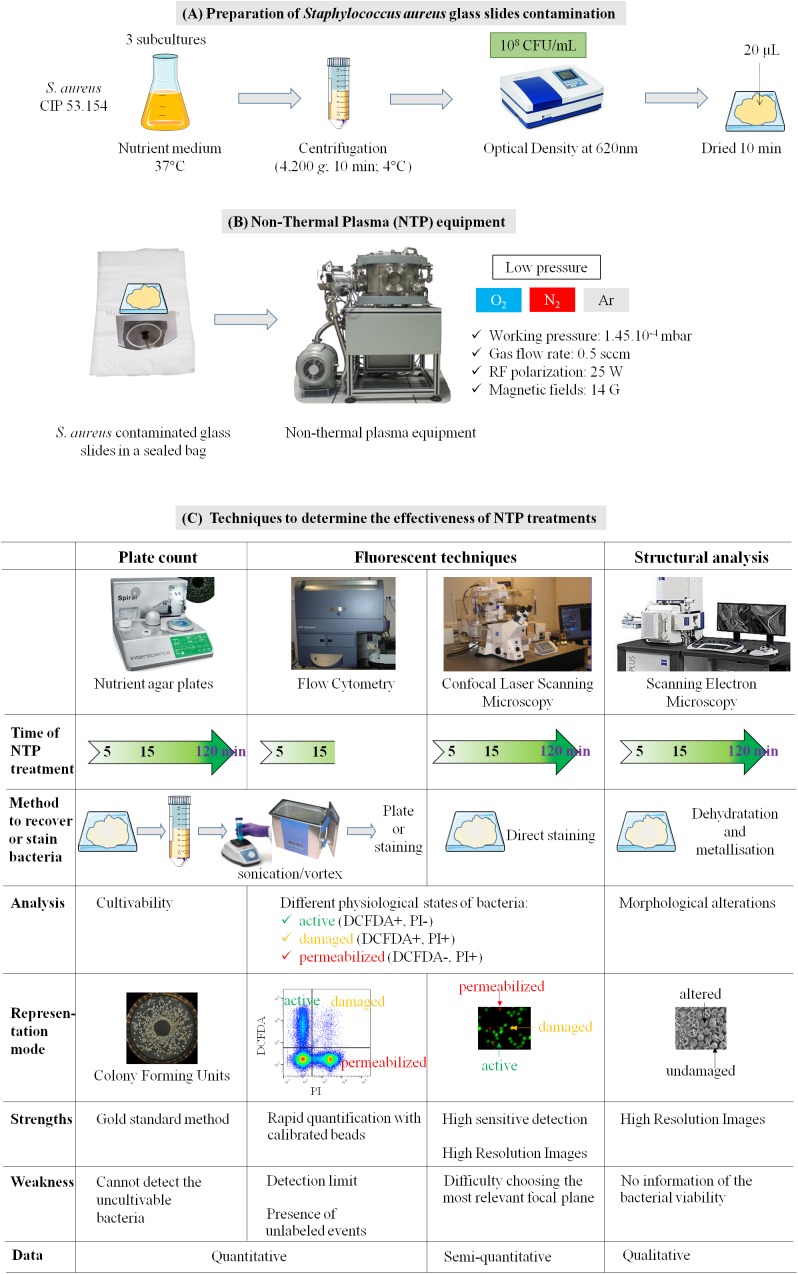
General flow chart of **(A)** preparation of contaminated glass slides; **(B)** NTP equipment; **(C)** techniques selected to determine the efficiency of NTP treatments in function to the duration of treatments.

Scanning electron microscopy method was used to observe the *S. aureus* surface after the different NTP treatments. Within the 5 min of O_2_ or Ar NTP treatments, remarkable morphological changes have been observed, which correlates with CLSM results. When *S. aureus* multilayers are present on the glass slides, only the upper layers of the bacterial deposit were damaged after 15 min of NTP treatments, while bacteria located in the deepest layers seemed morphologically intact. This bacterial organization close to a biofilm structuration is well known for its strong resistance to NTP treatments (Moreau et al., 2005; Kamgang et al., 2007; Joshi et al., 2010; Ermolaeva et al., 2011; Guo et al., 2015; Puligundla and Mok, 2017). After 120 min of NTP treatments, whatever the gas used, a 6 log reduction was obtained with the plate counting method, and only debris could be visualized by SEM. However, a few labeled bacteria were observed by CLSM even if O_2_ NTP treatment seemed to be the most efficient. **Figure 6** summarizes the techniques used to monitor and determine the efficiency of our NTP treatments up to 120 min.

The inactivation mechanism of NTP is still unclear. However, three mechanisms are usually proposed: UV radiations of genetic material, photodesorption of cell by UV radiations and etching by ions and chemical reactive species (Moisan et al., 2001, 2002; De Geyter and Morent, 2012; Fröhling and Schlüter, 2015).

In our low pressure NTP process, the reactive species of Ar, O_2_, and N_2_ and UV photons are generated to inactivate bacteria and presumably VUV photons too (Halfmann et al., 2007; Rauscher et al., 2010; Guo et al., 2015). However, some authors highlighted the main role of chemical active species in the inactivation compared to UV and VUV photons (Rauscher et al., 2010; Guo et al., 2015).

We have demonstrated previously that a short time of Ar NTP treatment was the most efficient to inactivate *S. aureus*. To explain this early efficiency between the plasmas, we could supposed that Ar^+^ could lead to an etching process causing perforations of bacteria membranes as suggested by Opretzka et al. (2007). When Ar–He plasma was applied, they observed perforations of *Bacillus atrophaeus* spore coat allowing reactive components from plasma to penetrate in the cell to act directly. Moreover, they suggested that it was a crucial point to alter multi-layers stacks of bacteria or bacteria embedded in a matrix.

After a longer time of exposure, O_2_ NTP treatment was the most efficient. O_2_ plasma is well-known to generate ROS, UV photons and probably metastable state O_2_^∗^ (Guo et al., 2015). Among to these reactive components, the ROS are involved in etching process and then to oxidative stress leading to the bacteria death.

Moreover, different authors have shown the interest of O_2_ and Ar mixture NTP treatment to inactive resistant microbes as spores or to remove bacterial protein residues (Raballand et al., 2008; Rossi et al., 2009; von Keudell et al., 2010). Etching and desorption mechanisms could implied in the permeabilization of bacteria membranes and specially of the characteristic peptidoglycan of Gram-positive bacteria. As a consequence, it can be suggested that if this permeabilization did not lead to the *S. aureus* death, VBNC could be generated.

## Conclusion

Our NTP process is a promising alternative to conventional technologies for innovative medical devices sterilization. However, to evaluate its efficiency, several precautions must be taken. Indeed, the plate counting method was not sufficient by itself to ensure that no bacteria can recover and grow. Hence, fluorescent methods by determining different physiological states have showed their interest to evaluate the efficiency of the NTP process. CLSM technique showed that a few active bacteria are still present downstream the NTP treatment. To improve the treatment efficiency, it would be interesting to test an Ar–O_2_ mixture NTP at different concentrations or alternate use of both Ar and O_2_ plasmas. Additionally to optimize our sterilization technique, we will identify the generated particles from our NTP process and then will investigate their bactericidal mechanisms.

## Author Contributions

MB and M-PG contributed to the prototype conception. GC, EC, CD, ZBB, and M-PG contributed to the bacterial studies. GC, EC, and M-PG contributed to the conception of the work. GC and EC performed the fluorescent experiment. GC, SA, and CT analyzed the cytometric and confocal data. GC, SA, CT, SG, and M-PG wrote the manuscript. All authors read and approved the final manuscript.

## Conflict of Interest Statement

The authors declare that the research was conducted in the absence of any commercial or financial relationships that could be construed as a potential conflict of interest.
