# Supplementary material for: Contribution of Fluorescence Techniques in Determining the Efficiency of the Non-thermal Plasma Treatment
Source: Front Microbiol. 2018 Sep 10;9:2171. doi: 10.3389/fmicb.2018.02171 (PMC6140754; doi:10.3389/fmicb.2018.02171)
Supplement: Supplementary file 1 [file Data_Sheet_1.docx]

Supplementary Material

Contribution of Fluorescence Techniques in Determining the Efficiency of the Non-Thermal Plasma Treatment

Gaëlle Carré, Emilie Charpentier, Sandra Audonnet, Christine Terryn, Mohamed Boudifa, Christelle Doliwa, Zouhair Ben Belgacem, Sophie C. Gangloff, Marie-Paule Gelle*

*** Correspondence:** Corresponding Author: marie-paule.gelle@univ-reims.fr

| (A)   | (B)   |
| --- | --- |
| (C)   | (D)   |

**Supplementary Figure 1.** Concentration per mL of **(A)** total labeled events; **(B)** active population (DCFDA+ PI-); **(C)** damaged population (DCFDA+ PI+) and **(D)** permeabilized population (DCFDA- PI+) determined by flow cytometry after exposure of *S. aureus* to low pressure or after 5 or 15 min of NTP treatments (n=7). Statistical analysis with the Wilcoxon test p <0.05 (*); p <0.01 (**).

| (A)   | (B)   |
| --- | --- |
| (C)   | (D)   |

**Supplementary Figure 2.** Density of *S. aureus* population determined by CLSM with DCFDA and PI after O_2_, Ar and N_2_ plasma treatments for 5 and 120 min (events/mm^2^). Evaluation of the total density in events/mm^2^ of: **(A)** total labeled events; **(B)** active bacteria (DCFDA+ PI-); **(C)** damaged bacteria (DCFDA+ PI+); **(D)** permeabilized bacteria (DCFDA- PI+) (n≥3). Statistical analysis with the Wilcoxon test p<0.05 (*); p<0.01 (**); p<0.001 (***).
